# Immune Cells in Head-and-Neck Tumor Microenvironments

**DOI:** 10.3390/jpm12091521

**Published:** 2022-09-16

**Authors:** Enar Jumaniyazova, Anastasiya Lokhonina, Dzhuliia Dzhalilova, Anna Kosyreva, Timur Fatkhudinov

**Affiliations:** 1 Department of Histology, Cytology and Embryology, Peoples’ Friendship University of Russia (RUDN University), 6 Miklukho-Maklaya Street, 117198 Moscow, Russia; 2 National Medical Research Center for Obstetrics, Gynecology and Perinatology Named after Academician V.I. Kulakov of Ministry of Healthcare of Russian Federation, 4 Oparina Street, 117997 Moscow, Russia; 3 Avtsyn Research Institute of Human Morphology of Petrovsky National Research Centre of Surgery, 3 Tsyurupy Street, 117418 Moscow, Russia

**Keywords:** head-and-neck tumors, HNSCC, tumor microenvironments, tumor-associated macrophages, B cells, regulatory T cells, immunotherapy

## Abstract

Head-and-neck cancers constitute a heterogeneous group of aggressive tumors with high incidence and low survival rates, collectively being the sixth most prevalent cancer type globally. About 90% of head-and-neck cancers are classified as squamous cell carcinomas (HNSCC). The innate and adaptive immune systems, indispensable for anti-cancer immune surveillance, largely define the rates of HNSCC emergence and progression. HNSCC microenvironments harbor multiple cell types that infiltrate the tumors and interact both with tumor cells and among themselves. Gradually, tumor cells learn to manipulate the immune system, either by adapting their own immunogenicity or through the release of immunosuppressive molecules. These interactions continuously evolve and shape the tumor microenvironment, both structurally and functionally, facilitating angiogenesis, proliferation and metastasis. Our understanding of this evolution is directly related to success in the development of advanced therapies. This review focuses on the key mechanisms that rule HNSCC infiltration, featuring particular immune cell types and their roles in the pathogenesis. A close focus on the tumor-immunity interactions will help identify new immunotherapeutic targets in patients with HNSCC.

## 1. Introduction

The term ‘head-and-neck tumors’ refers to malignant neoplasms of diverse histological grade and structure, derived from oropharynx, tonsils, lower pharynx, mobile tongue, oral and nasal structures, paranasal sinuses or salivary glands [1]. The majority of these tumors, arising from mucous epithelia of the mouth, pharynx or larynx, are classified as head-and-neck squamous cell carcinoma (HNSCC). Oral and laryngeal cancers have been associated with tobacco/alcohol use, whereas pharyngeal cancers tend to involve human papillomavirus (HPV), notably the oncogenic HPV-16. In this regard, HNSCCs are classified into HPV-negative and HPV-positive. The test for HPV status in patients with HNSCC is pivotal for the choice of treatment strategy and prognosis [2,3]. HNSCC is often diagnosed at advanced stages because of asymptomatic its course and the lack of pre-cancer lesions [4]. The choice of therapy depends on the clinical stage as the major risk factor; the traditional tumor/[lymph] nodes/metastasis (TNM) staging system has been complemented by the AJCC/UICC update of 2017 conveying additional information with regard to HPV-positive disease [5,6]. Despite the extensive research and development into new therapies, 5-year survival rates with HNSCC remain low due to late diagnosis, metastatic/recurrent course and therapy resistance of the disease [7]. The first-line options of surgical resection and radiation therapy often fail to prevent progression; the situation is aggravated by high incidence of chemoradiotherapy resistance in HNSCC [8]. To date, major research efforts are concentrated on understanding HNSCC biology, notably immunobiology, in a pursuit of prognostic, diagnostic and therapeutically targetable candidate markers for the development of advanced efficacious methods of lower toxicity and higher specificity. Molecular studies of genetic landscapes and aberrant signaling pathways are known to provide multiple cues to pathogenesis and help produce detailed classifications of tumors based on genetic and epigenetic signatures. For HNSCC, an extensive body of molecular-genetic data has been accumulated, requiring accurate biological interpretation. It has also become evident that HNSCC has high intrinsic rates of mutagenesis that create neoantigens and certainly call for immunotherapeutic options [9,10]. Recent trials of new immune checkpoint inhibitors may forerun a paradigm shift in head-and-neck cancer therapies [8,11,12].

## 2. The Role of Immune Cells in Tumor Microenvironments

Our current understanding of the role of immune system in tumorigenesis is a remarkable achievement of fundamental medicine.

In 1957, Lewis Thomas and Frank MacFarlane Burnet proposed the immunological surveillance concept explicitly featuring for the first time the role of immunity in cancer. According to this concept, the immune system is endowed with the ability to restrict tumor growth through recognition of antigens expressed by pre-cancerous cells and eliminate these cells well in advance of the possible transition to clinical disease [13].

The sentinel role of the immune system in the endogenous prevention of malignant neoplastic processes is supported by the dramatically increased incidence of various cancers (lymphomas, gastric, breast, bladder, cervical cancers, etc.) among patients with immunodeficiencies [14]. On the other hand, solid tumors (which represent a catastrophic failure of such endogenous prevention) are commonly infiltrated with various and abundant immune cells, dynamically interacting with the tumor microenvironment (TME) [15]. The role of the immune system in cancer is dual. Indeed, robust and timely responses orchestrated by tumor-specific immunocompetent cells can significantly suppress tumor growth and propagation in the body. However, there emerges an opposite trend, which may, at a certain breaking point, become mainstream; under the negative, counter-educating influence of the TME, immune cells become effectively converted into tolerogenic subtypes capable of potently supporting the disease [16,17].

Until recently, the fundamental research on head-and-neck carcinogenesis almost entirely focused on tumor cell morphology, protein expression, mutational profiles, etc. However, the aggressive clinical course of HNSCC complicated by the immunosuppressive capacity of these tumors and accompanied by radiochemotherapy, and occasionally also immunotherapy, resistance underscores their dynamic complexity irreducible to the properties of tumor cells per se. The research focus has, therefore, been expanded to involve the TME, notably infiltrating immunocompetent cells. An increasing number of recent studies demonstrate pivotal roles of tumor microenvironments in the nucleation, progression, invasion and therapy resistance of a tumor [11,17,18].

**Figure 1:**
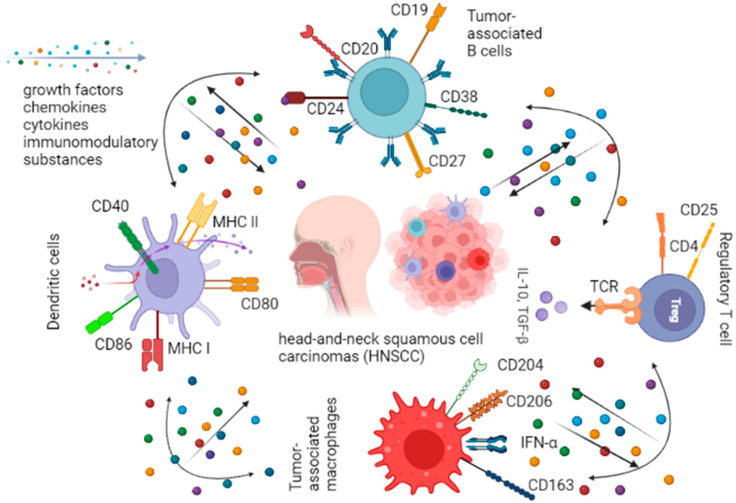
Immune cells in head-and-neck tumor microenvironments.

Cellular components of the TME include immune cells (of hemopoietic origin), fibroblasts, mesenchymal cells, vascular endothelial cells and nerve fibers; incidentally, TME cells often exceed the tumor cells by count (Figure 1) [18]. Apart from its cellular component, the TME contains the fibrous matrix produced by its cells and is rich in diverse growth factors, nutrients, metabolites and intermediates, hormones and paracrine factors, including chemokines, cytokines and immunomodulatory substances, secreted jointly by the tumor and its stroma. These factors support positive selection of aggressive clones and promote further malignization [19] making the TME a favorable, permissive milieu for tumor progression, metastasis and therapy resistance.

By default, the healthy (non-transformed) cells and structures of the body, occurring in the TME, might be expected to play a protective role, limiting tumor growth and its propagation by metastasis in every possible way. However, the reality involves the enormous selective pressure fed by unrestricted cell growth and continual leukocyte infiltration of the tumor. Eventually, under the influence of specific paracrine cocktails produced by the tumor, the immune cells in the TME succumb and turn against the body, facilitating tumor progression and favoring its growth.

Noteworthy, the cellular composition of the TME in head-and-neck tumors depends on etiological factors, notably the HPV status; in connection with these differences HPV+ and HPV− patients show different responses to therapy [2,16].

The normal immune functionalities demand a concerted action of the innate and adaptive immune systems. The innate immune system, with its immediate local reactions to foreign agents, ensures primary deterrence of the pathogen while providing a reliable physiological substratum for the development of adaptive immune responses required for the final clearance and immunological memory generation. The antigen-presenting cells (APC), which include monocytes, macrophages, B cells and dendritic cells, are central to the entire scheme as they provide the vital link between the innate and adaptive immunity reactions [1,20]. 

In recent years, the role of the immune system in the growth and progression of head-and-neck tumors has been extensively studied worldwide. The studies reveal a connection of lymphocytic infiltration with prognosis, with a high content of tumor-infiltrating lymphocytes (notably CD8+ cells) in head-and-neck tumors being a favorable predictor.

However, the role of the immune system is never unconditionally beneficial for the body; the net output depends on physiological context. This ambiguity is especially evident for particular cell types. For example, regulatory T cells exert control over autoreactive lymphocytes, but also inhibit immune responses to tumor-associated antigens. High counts of regulatory T cells observed within head-and-neck tumors and even in the peripheral blood of the patients [12] indicate the pronounced immunosuppressive effect of head-and-neck tumors exerted, not only locally but also, at the systemic level. 

On the other hand, infiltrations with T cells polarized towards a cytotoxic immune response have been associated with lower rates of recurrence. Extended to a wide variety of human cancers, these observations position the intratumoral cytotoxic T cell infiltration as a strong predictor.

The modifying effects of tumors on host immunity has been termed ‘immunoediting’. Initially developed by Dunn et al. [21], the immunoediting theory underscores the dual role of the immune system in tumor progression, defining the crosstalk between immune and malignant cells as a very fine dynamic interaction that proceeds in three distinct phases: elimination, equilibrium and escape.

## 3. Dendritic Cells

Dendritic cells (DC) constitute a heterogeneous population of motile antigen-presenting cells classified into several subtypes differing by ontogeny, localization and phenotype [20]. Each population of DC has its distinct way of interacting with T, B and NK cells reflected by specific profiles of receptor expression and cytokine production [20,22]. 

From a functional standpoint, DC are considered the most potent professional APC, which engulf, process and present various peptide modules, including tumor-associated epitopes, to the naïve antigen-specific T cells [17,23].

DC originate from CD34+ bone marrow progenitors and migrate with circulation to peripheral non-lymphoid tissues where they capture antigens; eventually, DC move to lymphoid tissues where the captured antigens are processed and presented to T cells. In the TME of head-and-neck tumors, myeloid and plasmacytoid DC are distinguished, which plays a special role in the tumor-associated immune reactions [24]. Each subpopulation of DC exerts its own spectrum of immunomodulatory effects: myeloid DC are known to augment immune responses (anti-tumor or anti-inflammatory), whereas lymphoid and plasmacytoid subtypes support immune deterrence and/or tolerance [23,25].

As was stated earlier, DC are able to induce anti-tumor T cell responses. Upon capturing tumor antigens, DC present the fragments at their surface, thus, activating differentiation of the naïve CD4+ and CD8+ T cells into tumor-specific effector T cells [24,26,27]. Consistently, the abundance of DC in head-and-neck the TME has been associated with a favorable prognosis [28,29]. On the other hand, the inhibitory effect of a tumor on differentiation and functional activity of tumor-associated DC may lead to pathological alterations in their properties as assessed in vitro and in vivo [27]. These tumor-induced changes in DC profiles are considered essential prerequisites to the impaired anti-tumor immunity and have low efficacy with various treatment modalities. Each subtype of DC in the TME of head-and-neck tumors will be described in more detail.

Myeloid DC are the predominant category of DC and express high levels of MHC class II, costimulatory molecules (CD40, CD80 and CD86) and adhesion molecules (CD11a, CD15s, CD18, CD29, CD44, CD49d, CD50 and CD54), which are necessary for immunological synapse development and T-lymphocyte activation [30,31]. However, cancer cells often succeed in producing various factors that inhibit normal functioning of DC. Such factors may stimulate dendritic cell apoptosis; alternatively, they may interfere with dendritic cell maturation. As the programmed death of DC is an important regulatory factor of the length and strength of immune responses, a decline in dendritic cell components in the TME renders the induction of anti-tumor immune responses inefficient, thus, facilitating tumor escape from recognition and elimination by the immune system [24].

One study [32] confirms a favorable prognosis in patients with head-and-neck tumors presenting with a high content of CD3+ and CD8+ T cells in the TME. Other studies [24,30] provide additional evidence that a high content of myeloid DC in the head-and-neck TME correlates with high counts of tumor-infiltrating lymphocytes and contributes to a favorable prognosis and a good anti-tumor therapy response.

Apart from the myeloid DC, head-and-neck TMEs contain plasmacytoid dendrite cell subpopulations [29,31]. The available data suggest a dual, controversial role of plasmacytoid DC in the anti-tumor immunity: the cells initiate immune responses toward the malignant tumor while, simultaneously, inducing a state of immune tolerance which promotes its progression [33].

Plasmacytoid DC morphologically resemble secretory lymphocytes and plasma cells. A distinctive feature of plasmacytoid DC is the presence of Toll-like receptors of types 7 and 9. Normally (if no foreign agents have entered the body), plasmacytoid DC circulate in the blood and are not detected in peripheral tissues. In response to the appearance of foreign DNA-antigen, they (plasmacytoid DC) can migrate to the site of the damage and begin active synthesis of high doses of IFN- I, significantly contributing to the protective function of the immune system [23].

In head-and-neck TMEs, DC are predominantly concentrated in layers of connective tissue adjacent to the tumor. Plasmacytoid DC usually promote the anti-tumor responses via the Toll-like receptor 9 (TLR9) -dependent synthesis of interferon-α (IFN-α), interleukin-6 (IL-6) and tumor necrosis factor-α (TNF-α). Noteworthy, the plasmacytoid DC are infiltrated with decreased production levels of IFN-α, TNF-α and IL-6 which have been identified in oral squamous cell carcinoma and have been associated with decreased overall survival [34,35].

A suppressive effect of tumor cells on the production of IFN-α by plasmacytoid DC has been reported [36]. Specifically, the tumors are able to inhibit TLR-9 [36]; thus, although plasmacytoid DC enter the TME, their ability to promote anti-tumor responses inside the tumor is compromised. It has also been suggested that, in the absence of appropriate stimulation, plasmacytoid DC promote tolerance by inducing and supporting regulatory T cells [35]. Consistently, increased counts of plasmacytoid DC correlate with increased tumor size, lymph node metastasis and adverse clinical outcomes in HNSCC [34].

Plasmacytoid DC collected from tumors show high expression of immunosuppressive molecules, including indoleamine 2,3-dioxygenase 1 (IDO1), programmed death ligand 1 (PD-L1), IL-10 and FAS ligand, which help plasmacytoid DC induce apoptosis or anergy of activated T cells, as well as their transition into regulatory T cells [16].

Their activatory connection to the cellular link of immunity, along with the high antigen presentation efficiency, allows distinguishing DC as a candidate target of anti-tumor therapy among APC. With regard to their evident role in anti-tumor immunity, the possibility of using DC in the treatment of various types of malignant neoplasms is being extensively studied [23].

The most important achievements in this field include the development of methods for obtaining DC from peripheral blood monocytes and bone marrow progenitors ex vivo, which significantly expands the research opportunities and stipulates the clinical use of DC vaccines [37], although the applicability of such vaccines to head-and-neck cancers is disputable [27].

## 4. Tumor-Associated Macrophages

Macrophages play prominent roles in both innate and adaptive immunity reactions. These cells, endowed with phagocytic activity and antigen-presenting capacity, are differentiated from blood monocytes upon their transfer to peripheral tissues through the circulation. Macrophages constitute cell populations of enormous plasticity, capable of trans-differentiation between phenotypes [38,39]. This process, widely known as ‘macrophage polarization’, is highly reflective of the conditions and kind of stimuli the cells are exposed to at a particular time point. The local cytokine milieu is one of the key factors in macrophage polarization. Extensive studies on expression of specific markers and production of biologically active substances by macrophages in mammals (humans, mice and rats) identify two major macrophageal subtypes conventionally denoted as the ‘classically activated’ (i.e., pro-inflammatory, M1) and the ‘alternatively activated’ (i.e., anti-inflammatory, M2) [40,41,42]. According to this paradigm, macrophages can switch their phenotypes in response to microenvironmental stimuli; importantly, in the absence of relevant external stimuli, the cells maintain their naïve state M0 [43]. Macrophages usually polarize into M1 phenotype in response to pro-inflammatory cytokines, e.g., IFN-γ and TNF-α, as well as pathogen-related stimuli, e.g., lipopolysaccharide. The M1-polarized macrophages produce TNF-α, IL-1, IL-6, IL-12, IL-23, chemokine CCL8, monocyte chemotactic protein 1 (MCP-1), macrophage inflammatory protein 2 (MIP-2), ROS, cyclooxygenase 2 (COX-2), CD16 and CD32, as well as the inducible NO synthase (iNOS), expression of which marks M1 polarization in rodents. This macrophage subpopulation is predominantly involved in pro-inflammatory reactions, including chemotaxis, free radical production and extracellular matrix degradation, as well as anti-microbial and anti-tumor activities [44]. By contrast, the M2-polarized macrophages are derived from naïve cells by exposure to anti-inflammatory stimuli (Th2 cytokines IL-4 and IL-13, IL-10, TGF-β, etc.) and participate in anti-inflammatory reactions [45]. The M2-polarized macrophages express high levels of IL-1 receptor antagonist (IL-1RA), arginase 1 (Arg-1), other anti-inflammatory cytokines and chemokines (IL-10, TGF-β, CCL18, etc.) and low levels of IL-12. These cells suppress effector T cells and promote tissue repair and wound healing by secreting vascular endothelial growth factor (VEGF), epidermal growth factor (EGF), platelet-derived growth factor (PDGF) and IL-10; they also participate in Th2-reactions and extracellular matrix remodeling [45,46,47,48].

The term ‘tumor-associated macrophages’ (TAM) commonly refers to mature macrophages found in the TME. The vast majority of TAM are M2-polarized macrophages.

The recruitment of macrophages to the TME may involve fresh bone marrow-derived cells or be proceed by differentiation of myeloid-derived suppressor cells in the TME. The process is regulated by several hemopoietic growth factors including CSF1, MCP-1 and chemokines. The hypoxic conditions characteristic of TMEs favor the assimilation of TAM in the TME [49,50]. Upon assimilation, TAM vigorously promote the creation of an immunosuppressive milieu by a variety of mechanisms including the metabolic repression of T cells, expression of PD-L1 and other surface molecules, suppression of NK cells and the synthesis of immunosuppressive cytokines, notably IL-6, IL-10 and TGF-β [50].

Multiple clinical studies indicate that TAM counts positively correlate with poor prognoses [51]. High macrophage numbers in the TME are considered an adverse prognostic factor in various solid tumors, e.g., breast, gastric and ovarian cancers [52,53,54]. It should be noted that elevated production of macrophage growth factors and chemokines by such tumors represents an adverse prognostic factor on its own. For example, β-chemokine CCL2 is often produced by ovarian, cervical, bladder, breast and brain cancers and its elevated expression indicates a poor prognosis specifically in breast, cervical and bladder tumors [55,56,57]. Another such molecule, cytokine M-CSF, responsible for macrophage survival and differentiation, is secreted by ovarian, uterine, breast and prostate tumors [58,59,60] and its elevated expression invariably correlates with a poor prognosis independent of the tumor localization [58,59]. In addition, the elevated expression of M-CSF by breast cancers has been associated with increased rates of macrophage infiltration in 90% of the cases [61]. Thus, clinical studies fully corroborate the suggestion that TAM thrive in rapidly progressing solid tumors amid a favorable spectrum of chemokines and growth factors, generally supporting the tumorigenesis.

Participation of TAM in HNSCC is an established fact: high TAM counts in the TME correlate with lymph node metastasis and advanced stages of HNSCC [43,48,62,63]. It has also been demonstrated that the TME in HNSCC contains predominantly M2-polarized TAM amid elevated levels of TGF-β [64]. A retrospective immunohistochemical study of TAM in biopsies collected from patients with oral cavity squamous cell carcinoma (OSCC) revealed high expression levels of anti-inflammatory IL-10 and TGF-β cytokines characteristic of M2-like phenotypes [64], along with elevated levels of soluble IL-10 and TGF-β in OSCC compared with matching biopsies of normal oral mucosa [64]. Similarly, TAM counts were associated with metastatic disease [64] and unfavorable outcomes [65,66], [14,67,68] in OSCC by several retrospective studies. Conversely, the highest macrophage counts in the biopsies of non-smoking/non-drinking patients with OSCC [69] correlated with a lack of cancer stem cells markers, NANOG and SOX2, along with high expression of PD-L1 by these tumors, indirectly pointing to the role of macrophages in immune escape [69]. Another study revealed a significant correlation of TAM counts with the expression of cancer stem cell markers SOX2 and ALDH1 [65]. A possible association of TAM with the epithelial-mesenchymal transition in OSCC was also demonstrated [62]. In a study of surgical biopsies from patients with low-grade dysplasia and OSCC, the macrophage content increased with the cancer progression; of note, TAM were especially abundant in HPV-positive tumors [65]. 

## 5. Tumor-Associated B Cells

The principal function of B cells is the implementation of humoral immunity reactions through interactions with T helpers and DC. B cell infiltrations have been observed in various cancers (lung, ovarian, liver, skin, cervical, rectal, prostate and head-and-neck) and characterized as a favorable prognostic factor positively correlating with survival [70,71,72,73,74,75,76]. Progression of such tumors has been shown to depend on the interactions of B cells with T helpers [77].

B cells constitute about 25% of all immune cells infiltrating the tumor nodules [37]. Their surface antigens, which include programmed cell death protein 1 (PD-1) and its ligand PD-L1, cytotoxic T-lymphocyte-associated protein 4 (CTLA-4) and the antigen-presenting cell integral membrane protein B7, provide substratum for the development of targeted immunotherapies for head-and-neck cancers directed towards correction of the tumor-associated B cell functionalities, [11,78,79,80]. At the same time, the impact of B cells on the efficiency of anti-tumor therapies remains uncertain.

In head-and-neck cancers, accumulations of the infiltrating B cells can be observed at the boundary with normal tissues, in tertiary lymphoid structures and, occasionally, within the tumor nodules. B cell infiltrates can inhibit tumor growth by several mechanisms including immunoglobulin secretion, activation of T cell responses and the antibody-dependent direct cytotoxic effects on tumor cells [81]. According to a meta-analysis by Wouters and Nelson [82], the counts of tumor-infiltrating CD20+ B cells, along with plasma cells, correlate with favorable prognoses in ovarian and breast cancers, hepatocellular cancers and sarcomas, esophageal and biliary cancers, whereas in non-small cell lung cancers, colorectal cancers and HNSCC the prognostic value of B cell infiltration rates remains disputable. The controversy may be related to the apparent heterogeneity of cohorts and tumors, as well as the diversity of immunocompetent cell repertoires in the TME [83]. Microenvironments of tumor nodules in HNSCC reveal an increased content of CD86+-activated and CD86+CD21-antigen-presenting B cells, as well as IgD-CD27+ memory B cells [83]. Lechner et al. [76] reported increased counts of CD27+/CD38hi/CD20- plasmoblasts in the TME and among peripheral blood mononuclear cells of patients with HNSCC. Pretscher et al. [84] reported a correlation of CD20+ B cell counts in HNSCC lymph node metastases with survival.

The tertiary lymphoid structures (TLS) are transient ectopic lymphoid aggregates resembling secondary lymphoid organs by their microanatomy and functions [85]. TLS are formed in response to chronic inflammation or infection [85,86] and comprise T- and B-dependent zones harboring differentiation of T and B effector cells, DC and memory cells [87,88]. TLS are known to be a source of tumor-infiltrating cytotoxic T cells, which relates them to the anti-tumor response potentiation and, ultimately, to survival [85,89]. Mature TLS have germinal centers containing B cells that express CD38 glycoprotein (CD38+IgD-) and BCL6 transcription factor [90]. According to Ruffin et al. [91], patients with HPV-positive HNSCC exhibit an increased volume of TLS with germinal centers across the affected area (intra- and peri-tumorally and outside the tumor nodules). Importantly, the degree of TLS development correlates with survival [91], which underscores the role of humoral immunity in the anti-tumor responses.

Immunocompetent cells of the TME in head-and-neck tumors, notably HNSCC, comprise a subpopulation of CD24hi/CD38hi/CD19+ regulatory B (Breg) cells, which predominate numerically over conventional CD19+ B cells and tend to occupy the ‘borderline’ niches at the boundary of the tumor with normal tissue [76]. These CD24hi/CD38hi/CD19+ Breg cells can reduce the degree of autoimmune reactions by inhibiting Th1 and Th17 cell differentiation while facilitating the development of regulatory T cells [92]. The adenosine-producing CD39+CD73+ Breg cells in HNSCC TME were shown to inhibit the effector B cell functionalities thus supporting immunosuppression [93]. Furthermore, CD19+IL10+ Breg cells in the TME of lingual squamous cell carcinomas promoted the differentiation of CD4+ T cells into regulatory T cells, correlating with adverse prognoses [92]. At the same time, the presence of atypical memory B cells (CD27-IgM-IgD-) and CD24hi/CD38hi Breg cells in lymph nodes remote from the tumor nodules in patients with HNSCC correlated with a low histological grade of the tumors and less pronounced infiltration of sentinel lymph nodes, possibly regarded as favorable prognostic indicators [94]. The obvious controversy of these findings reflects the differential prognostic value of Breg cells depending on their phenotypes and localization.

Thus, effector B cells, plasmoblasts and plasma cells found in the infiltrates and TSL augment the anti-tumor immunity by multiple routes that involve antibody production, antibody-dependent cytotoxicity, antigen presentation and activation of T cell immunity. However, it should be noted that Breg cells also present in the TME are capable of producing immunosuppressive cytokines (IL-10, TGF-β, etc.) which support tumor progression [95].

## 6. Regulatory T Cells

Regulatory T (Treg) cells constitute less than 5% of CD4+ T cells in peripheral circulation. Apart from CD4, these cells express CD25 antigen and forkhead box P3 (FOXP3) transcription factor. Treg cells are subdivided into natural Treg cells that mature in the thymus and peripheral Treg cells, i.e., induced or adaptive Treg cells, depending on their origin, localization and phenotypes. These cells play a key role in orchestrating the immune escape by producing immunosuppressive cytokines IL-10 and TGF-β while sponging IL-2. Thus, Treg cells express the negative costimulatory inhibitors of immune checkpoints, notably CTLA-4 and PD-1, which suppress the activity of T cells through binding with corresponding ligands [96].

At the same time, Treg cells are the principal immunosuppressive T cells in head-and-neck tumor microenvironments. Their inhibitory effects on effector T cell proliferation and activity are known to modulate immunotherapy-induced responses [18,97]. Treg cells implement their regulatory functions by controlling self-reactive lymphocytes and promoting their elimination on one hand, but inhibit the immune responses to tumor-associated antigens on the other hand. High contents of Treg cells in the TME, along with their elevated differential counts in peripheral blood, in patients with head-and-neck cancers have been reported [12,18,98] indicating, once again, that the profound immunosuppressive effects of head-and-neck cancers ascend to the systemic level. Furthermore, peripheral Treg cells are liable to recruitment to the TME where their immunosuppressive capacities are bolstered by high local concentrations of TGF-β [16,22,99]. The recruitment of Treg cells is mediated by chemokines and associated receptors, e.g., CCL28-CCR10 and CXCL12-CXCR4.

T reg cells express high quantities of CTLA-4 which binds CD80 and CD86 on antigen-presenting cells thereby reducing their effector T cell activating capacity [100].

High counts of Treg cells in HNSCC have been associated with radiotherapy resistance. Of note, the radiation therapy per se can boost the recruitment of Treg cells to the TME, thus, mitigating the radiation-induced tumor death effect [101]. These data position Treg cell dynamics as candidate prognostic indicators in HNSCC.

Current opinions on the prognostic significance and meaning of Treg cell counts in HNSCC vary. Although most of the experts associate the high content of Treg cells with favorable prognoses [102,103,104], the opposite opinion has also been expressed [100]. This uncertainty necessitates further studies of the role of Treg cells in HNSCC progression.

## 7. Immunotherapy

The dual role of immune cells in tumorigenesis implicates a substantial impact of the immune system on head-and-neck tumor progression and explains the growing clinical interest in immunotherapeutic options. Immunotherapy is a remarkable, relatively new concept aimed at steady relief of tumor-induced immunosuppression, succeeded by counter-mobilization of the host immunity towards fighting the disease.

**Figure 2:**
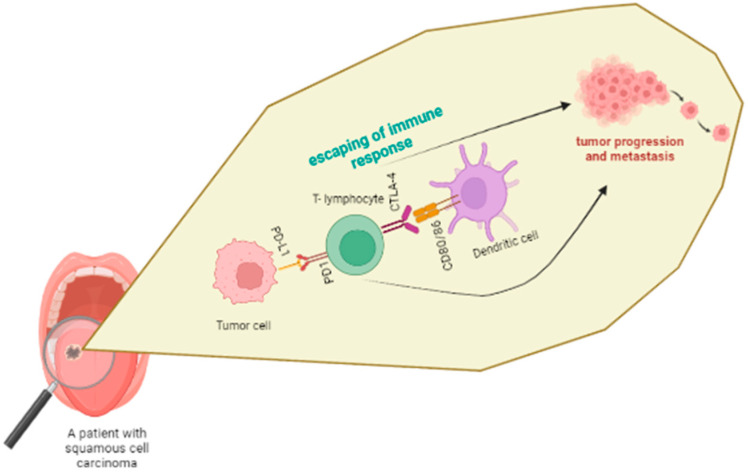
Escaping head-and-neck tumors from immune response.

The immune system normally recognizes tumor cells at a pre-cancer stage and eliminates them. However, the surviving tumor cells may eventually develop mechanisms preventing their recognition by the immune system and, thus, extinguish the response; this dynamic process, termed ‘immunoediting’, ultimately ensures the immune escape; Figure 2 [105].

Fundamental research on the interactions between HNSCC cells and the TME, and notably the immune escape mechanisms, provides the basis for the upgrade of existing clinical protocols with targeted immunotherapeutic options.

The availability of PD-1 antibodies significantly expands the clinical horizon for many tumors, including head-and-neck cancers. The immune checkpoint inhibitors pembrolizumab [106,107] (on its own or combined to chemotherapy) and nivolumab [108,109] (specifically for the platinum-resistant subtypes of head-and-neck cancers) are most heavily employed for this nosological spectrum. Despite certain side effects, these drugs effectively block the main route of immune escape for head-and-neck cancers: they make the tumor ‘visible’ to the host immune system and boost the chances of a favorable outcome. [11,110].

The immune checkpoint molecules, notably PD-1, its ligand PD-L1 and CTLA-4, are essential for self-tolerance, modulation of immune responses and maintenance of immune homeostasis in healthy tissues. Accordingly, the immune checkpoint inhibitors targeted at PD-1 (nivolumab and pembrolizumab), PD-L1 (atezolizumab, avelumab and durvalumab) and CTLA-4 (ipilimumab and tremelimumab) are capable of blocking these essential routes and thereby tightening up the immune surveillance over tumor cells. Dedicated clinical studies on the use of antibodies to PD-1, PD-L1 and CTLA-4 in patients with HNSCC [11] enable the bench-to-bedside translation of these drugs.

For decades, the epidermal growth factor receptor (EGFR) inhibitor cetuximab remained the only targeted drug approved for head-and-neck cancers. Advanced research on the mechanisms of immune escape laid the basis for a new generation of targeted drugs for HNSCC, e.g., the anti-PD-1 nivolumab and pembrolizumab or anti-PD-L1 atezolizumab [111]. Under normal conditions, the physiological PD-1 checkpoint inhibition regulates targeted T cell activation and anti-pathogen immunity, concomitantly preventing autoimmune reactions. When malignant transformation occurs, tumor cells try to employ these pathways to create an immunosuppressive milieu in order to protect themselves from elimination [112]. Thus, the immune escape harnesses the same mechanisms that normally schedule immune responses and control their intensity [113]. The block of PD-1/PD-L1-mediated immunosuppression permits the immune system to ‘re-educate’ itself, reconsider its attitude towards the tumor, identify it as a foreign entity and fight it properly [114,115].

However, head-and-neck tumors often develop immunotherapy resistance due to their huge mutational potential and high immunosuppressive capacity. This aspect requires urgent an update of the core principle with new strategies [6].

Pembrolizumab is an anti-PD-1 antibody, the efficacy and safety of which has been scrutinized in the KEYNOTE-012 multi-cohort study [116]. The drug is currently approved in more than 60 countries, including for the treatment of head-and-neck tumors [117,118].

The only immunotherapy drug approved for the treatment of metastatic HNSCC non-responding to platinum therapy is the PD-1 inhibitor nivolumab. In the meantime, many other immunotherapeutic options for HNSCC are currently being explored.

Several lines of research on immunotherapies are currently under way, notably combination therapies involving diverse immunotherapeutic approaches.

Compared with the use of individual checkpoint inhibitors, their combinations (e.g., the combined use of PD-1/PD-L1 and CTLA-4 antibodies) may afford substantial benefits. CTLA-4 is expressed at the surface of activated cytotoxic T cells and acts as a deterrent through binding the B7 ligands expressed on APC [119,120]. This binding inhibits the collateral activation of T cells in response to interactions between TCR and MHC [6]. The anti-PD-1/PD-L1/CTLA-4 combination immunotherapy is intended to exert an enhanced re-educating effect in head-and-neck cancers, as it simultaneously targets several routes of T cell suppression [113]. Several comparative clinical studies currently under way are focused on the combined use of ipilimumab and nivolumab in head-and-neck cancers. Overall, these studies reveal advantages of particular combinations of immune checkpoint inhibitors compared with other methods and modalities, notably the diminished side effects due to the highly selective biological action, especially considering that PD-L1 is specifically expressed by tumor cells.

Other candidate target molecules for drug-mediated inhibition in head-and-neck cancers include STAT3 (transcription factor augmenting the immunosuppressive potential of TME), LAG-3 (co-inhibitory surface receptor expressed by activated CD4+ and CD8+ T cells, as well as certain subpopulations of NK cells and DC; inhibits T cell activation) and IDO1 (inhibits T cell activation) [6].

The oncolytic virus therapies for head-and-neck cancers provide another promising option [121]. One study [122] revealed high sensitivity of HNSCC cells to an RNA-containing herpes simplex virus-based oncolytic vector in pre-clinical models. For instance, Telomelysin^®^ (also known as OBP-301) is a telomerase-specific and replication-capable oncolytic adenovirus that is effective against HNSCC. It causes tumor-specific replication and lysis in human cancer cells and acts as a radiosensitizer, inhibiting the repair of radiation-induced DNA damage. The oncolytic virus therapies reveal compatibility with standard chemotherapy regimens in phase I/II clinical trials [123]. Currently, there is active research on the possibility of combining therapy with oncolytic viruses and immunopreparations. A number of studies indicate very good compatibility of oncolytic virus therapy with immunopreparations. This may bring new perspectives in the field of antitumor therapy in the future.

Anti-cancer cell therapies involving T cells and DC constitute another emerging direction of translational research. Dendrite cell vaccines induce immune responses by boosting the numbers of tumor-infiltrating lymphocytes [23,27], whereas T cell infusions boost immunoreactivity [100,124]. The overall utility of such approaches in the clinical context of HNSCC remains uncertain: cell therapies definitely cannot be used on their own and, at the same time, imply repeated interventions (serial infusions of the ex vivo processed cell product). In addition, the in-host survival, homing efficacy and reproducibility of cell transplant behaviors once in the TME requires rigorous, dedicated research addressing multiple parameters because such transplants may support tolerances contrary to their therapeutic purpose [25,100,125,126].

The use of anti-HPV vaccines, possibly in combination with the basic anti-tumor regimens, is considered for HNSCC with a HPV-positive status. The options currently include MEDI0457 DNA vaccine (previously designated INO-3112 and containing two main components—INO-9012 plasmid encoding the immunity activator IL-12 and VGX-3100 plasmid with modified coding sequences of E7 and E6 viral proteins) or attenuated live vaccine ADXS11-001 as an alternative [124]. The efficacy of such vaccines in terms of stimulating T cell responses against endogenous antigens and their modifications that would be strong enough to induce the cytotoxic activity and flexible enough to account for the tumor heterogeneity [127] in head-and-neck cancers requires further investigation.

## 8. Conclusions

Immunological portraying of head-and-neck tumor microenvironments is highly relevant to the development of new therapies. For decades, high-quality research in this field has been focused solely on cancer cells and their mutations. However, it was the understanding of the dynamic interaction between cancer cells and their microenvironment that provided a clue to the mechanisms of tumor escape from immune surveillance and created a powerful impetus for the development of immunotherapy. The new drugs revolutionized the field of clinical oncology, particularly with regard to metastatic and/or radiochemotherapy resistant head-and-neck cancers. Meanwhile, the individual responses are complex and the regimen choices should be highly personalized on the basis of clinical data and a range of available targets and techniques. Currently, immunotherapies for head-and-neck cancers are at the dawn of their development.

Head-and-neck carcinomas use multiple mechanisms to escape from immune surveillance, which explains the futility of conventional attempts to interfere with their progression and metastasis. Understanding the complex relationship between the tumor and immune cells in its microenvironment, along with identification of cellular and molecular signaling pathways associated with the resistance to immune checkpoint inhibition, will advance the development of immunotherapeutic protocols for head-and-neck cancers.

## Data Availability

Not applicable.
